# Unsaturated Fatty Acid-Induced Conformational Transitions and Aggregation of the Repeat Domain of Tau

**DOI:** 10.3390/molecules25112716

**Published:** 2020-06-11

**Authors:** Carlo Giorgio Barracchia, Roberto Tira, Francesca Parolini, Francesca Munari, Luigi Bubacco, Georgios A. Spyroulias, Mariapina D’Onofrio, Michael Assfalg

**Affiliations:** 1 Department of Biotechnology, University of Verona, 37134 Verona, Italy; carlogiorgio.barracchia@univr.it (C.G.B.); roberto.tira@univr.it (R.T.); francesca.parolini@univr.it (F.P.); francesca.munari@univr.it (F.M.); mariapina.donofrio@univr.it (M.D.); 2 Department of Biology, University of Padova, 35131 Padova, Italy; luigi.bubacco@unipd.it; 3 Department of Pharmacy, University of Patras, 26504 Patras, Greece; G.A.Spyroulias@upatras.gr

**Keywords:** fatty acids, neurodegeneration, NMR spectroscopy, protein–lipid interactions, protein aggregation, Tau

## Abstract

Background: The intrinsically disordered, amyloidogenic protein Tau associates with diverse classes of molecules, including proteins, nucleic acids, and lipids. Mounting evidence suggests that fatty acid molecules could play a role in the dysfunction of this protein, however, their interaction with Tau remains poorly characterized. Methods: In a bid to elucidate the association of Tau with unsaturated fatty acids at the sub-molecular level, we carried out a variety of solution NMR experiments in combination with circular dichroism and fluorescence measurements. Our study shows that Tau^4RD^, the highly basic four-repeat domain of Tau, associates strongly with arachidonic and oleic acid assemblies in a high lipid/protein ratio, perturbing their supramolecular states and itself undergoing time-dependent structural adaptation. The structural signatures of Tau^4RD^/fatty acid aggregates appear similar for arachidonic acid and oleic acid, however, they are distinct from those of another prototypical intrinsically disordered protein, α-synuclein, when bound to these lipids, revealing protein-specific conformational adaptations. Both fatty acid molecules are found to invariably promote the self-aggregation of Tau^4RD^ and of α-synuclein. Conclusions: This study describes the reciprocal influence that Tau^4RD^ and fatty acids exert on their conformational states, contributing to our understanding of fundamental aspects of Tau/lipid co-assembly.

## 1. Introduction

Tau is an axonal cytosolic protein initially reported to promote the assembly of tubulin and to stabilize the microtubule network, thereby supporting neuronal cell function [1,2]. It is now becoming evident that the physiological role of Tau extends beyond its ability to modulate microtubule dynamics [3,4]. Indeed, the identification of numerous binding partners, including signaling molecules, cytoskeletal elements, and lipids, suggests its involvement in diverse activities [3]. The difficulty to precisely define the function of Tau is probably linked to the absence of a unique molecular structure as a consequence of its intrinsically disordered nature [5], its multiple post-translational modifications [6], and the occurrence of six isoforms [7].

**Scheme 1:**
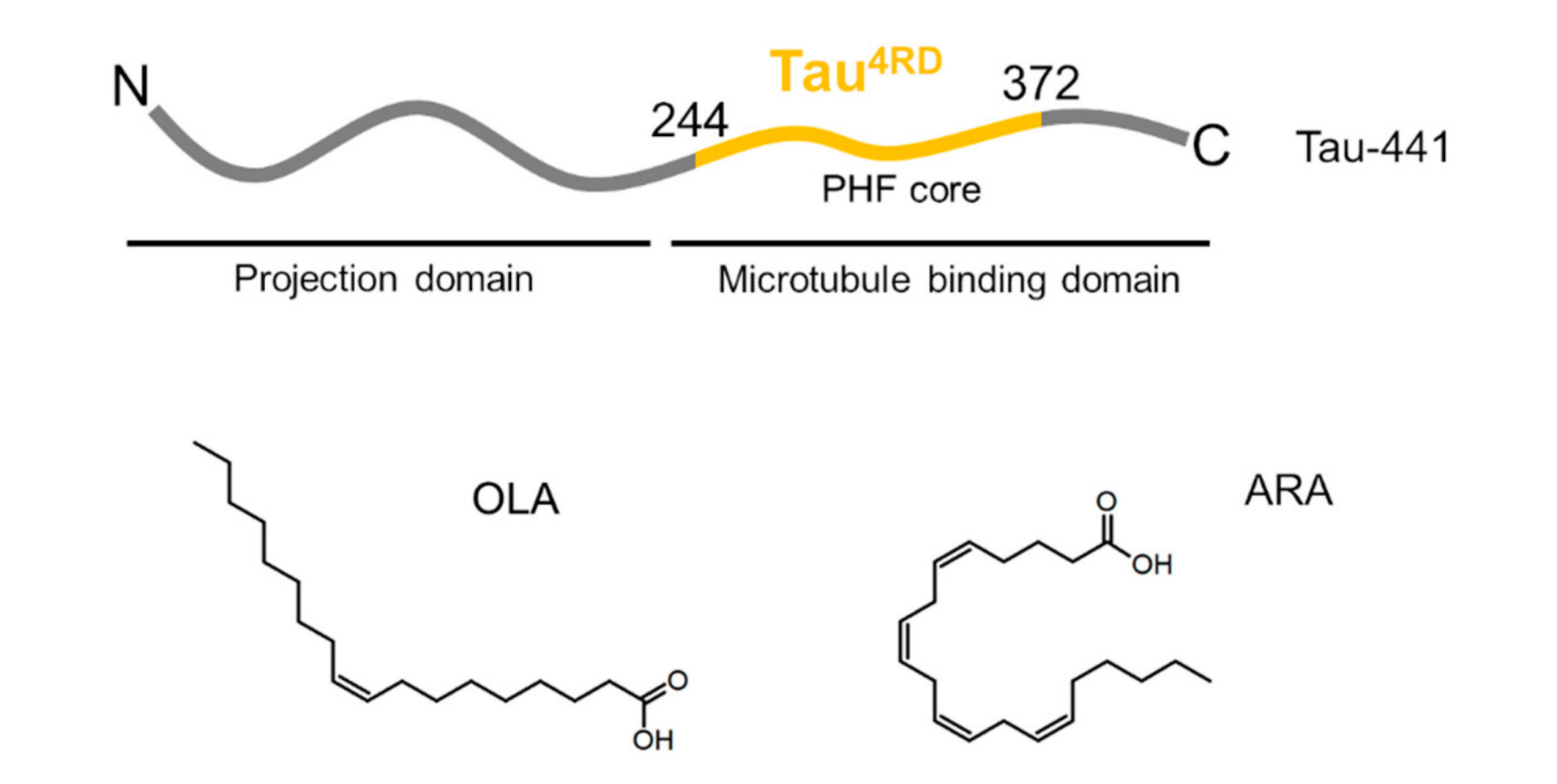
Illustration of Tau domain organization and the molecular structures of fatty acids.

The longest human Tau isoform (441 amino acids) is subdivided into two major domains (Scheme 1) [8]. The assembly domain in the carboxy-terminal part comprises the repeat domains and flanking regions; it is referred to as the microtubule binding domain. The projection domain is the amino-terminal section that projects away from microtubules. The amino acid sequence contains a low proportion of hydrophobic amino acids, consistent with the unfolded character of the protein. The four repeat domains exhibit a highly basic character due to an elevated number of lysine residues, which promote the association of Tau with microtubules. Two hexapeptide motifs PHF6* = ^275^VQIINK^280^ (in repeat R2) and PHF6 = ^306^VQIVYK^311^ (in R3) are predominantly hydrophobic and have propensity to form a β structure [9].

In its unbound state in solution, Tau is best described as a dynamic ensemble of rapidly interconverting conformers [10]. The dynamic structure of Tau is influenced by the presence of chemical modifications and the protein undergoes conformational rearrangement upon interaction with binding partners [11,12,13,14]. However, the most prominent conformational transition of Tau is the formation of supramolecular structures in a self-assembly process that eventually leads to the accumulation of insoluble deposits, containing hyperphosphorylated Tau, known as neurofibrillary tangles (NFTs) [15,16]. NFTs, constituted by paired helical filaments (PHF) and straight filaments (SF), are pathological hallmarks of a range of neurodegenerative disorders, including Alzheimer’s disease (AD), collectively referred to as tauopathies [17]. The pathological aggregation of Tau appears to be nucleated by the hexapeptide motifs and the repeat domains are part of the core of Tau filaments extracted from human tissues [18,19].

Remarkably, unmodified Tau has very little tendency to aggregate in solution. It is possible that a network of transient long-range contacts contributes to the prevention of protein self-assembly [10,20]. Indeed, truncated forms of Tau that lack these intramolecular interactions are more prone to aggregation [21] and the four-repeat domain, Tau^4RD^ (Scheme 1), is widely used as model polypeptide in aggregation studies. Point mutations that strengthen β-propensity and specific chemical modifications were reported to accelerate the fibrillization of Tau in vitro, however, the inducers of Tau aggregation in neurons and the structure of the aggregation-competent intermediate(s) remain currently unknown [22]. Anionic cofactors such as proteins, nucleic acids, and lipids were proposed to play a major role in shifting conformational equilibria towards either aggregation-resistant or pro-aggregating structures [22]. In vitro, sulfated glycosaminoglycans promote Tau aggregation through a charge compensation mechanism [23]. Similar effects are elicited by nucleic acids [24] and acidic lipids [25].

Lipids are abundant in the cellular environment and accessible to cytosolic Tau, and interactions between lipid membranes and Tau have been proposed to play a role in the protein’s physiological function [26,27], however, their influence on the conformational dynamics of Tau remains poorly characterized. Beyond plausible interactions under normal conditions, lipid-promoted Tau aggregation is of great interest due to its role in the process of neurodegeneration. It is known that the levels of free cytosolic fatty acids (FAs) can be increased in pathology [28]. Elevated concentrations of saturated FAs, which are able to cross the blood–brain barrier, constitute a significant risk factor for Alzheimer’s disease [29]. Brain neurons are particularly rich in polyunsaturated FAs, such as arachidonic (ARA) and docosahexaenoic (DHA) acids, which are liberated into the cytosol by the action of phospholipases [30]. Tau could participate in the formation of co-aggregates with lipids, in analogy with the formation of such assemblies by α-synuclein, another disordered amyloidogenic polypeptide, implicated in neurodegeneration [31,32]. This hypothesis is supported by the observation of Tau filaments associated with lipid membranes [33] and by the discovery of FAs in Alzheimer’s disease NFTs [34].

The importance of lipid–protein co-assembly in the onset and progress of amyloid diseases has been pointed out recently [35]. The association of intrinsically disordered polypeptides with free lipids, micelles, and vesicles is predicted to dramatically affect the structures and physicochemical properties of these biomolecules and to modulate their interactions with other molecular partners and cells [36,37]. Thorough investigations of lipid–protein co-assembly are necessary to deepen our understanding of both the healthy and aberrant action of amyloid proteins, providing the basis for the development of therapeutic strategies against devastating neurodegenerative diseases [38].

In our work, we aimed at elucidating the association of Tau with unsaturated FAs at the sub-molecular level. We focused on the interaction of Tau^4RD^ with arachidonic acid (ARA, all-*cis*-5,8,11,14-eicosatetraenoic acid), representing an abundant polyunsaturated FA in the brain, and oleic acid (OLA, *cis*-9-octadecenoic acid), a representative long-chain mono-unsaturated FA (Scheme 1). We applied a variety of solution NMR techniques, in combination with circular dichroism (CD) spectroscopy, to get insight into protein conformational transitions and dynamic supramolecular interactions. CD measurements were also performed on α-synuclein in the presence of FAs to be able to point out protein-specific behavior. FA-induced protein aggregation was monitored by thioflavin-T (ThT) fluorescence and transmission electron microscopy (TEM). We discuss our findings in light of current knowledge on Tau/lipid interactions.

## 2. Results

### 2.1. Aggregation State of Oleic and Arachidonic Acids in an Aqueous Solution

The dissolution of OLA and ARA in aqueous buffer solution results in the formation of supramolecular assemblies (e.g., micelles) in equilibrium with the monomeric species [39,40]. In the typical conditions of our experiments, the presence of such assemblies was apparent from ^1^H-NMR translational diffusion measurements. At a concentration of 75 μM, OLA was largely in aggregate form, as inferred from the lack of signal attenuation after a 200 ms diffusion period in pulsed field gradient NMR experiments (Figure S1A). At the same concentration, two distinct sets of signals were observed for ARA, one corresponding to fast diffusing molecules, another one to slowly diffusing large assemblies (Figure S1B). At higher concentrations, the slowly diffusing species were prevalent for both OLA and ARA (Figure S1C,D).

### 2.2. Association of Tau^4RD^ with Fatty Acids

**Figure 1:**
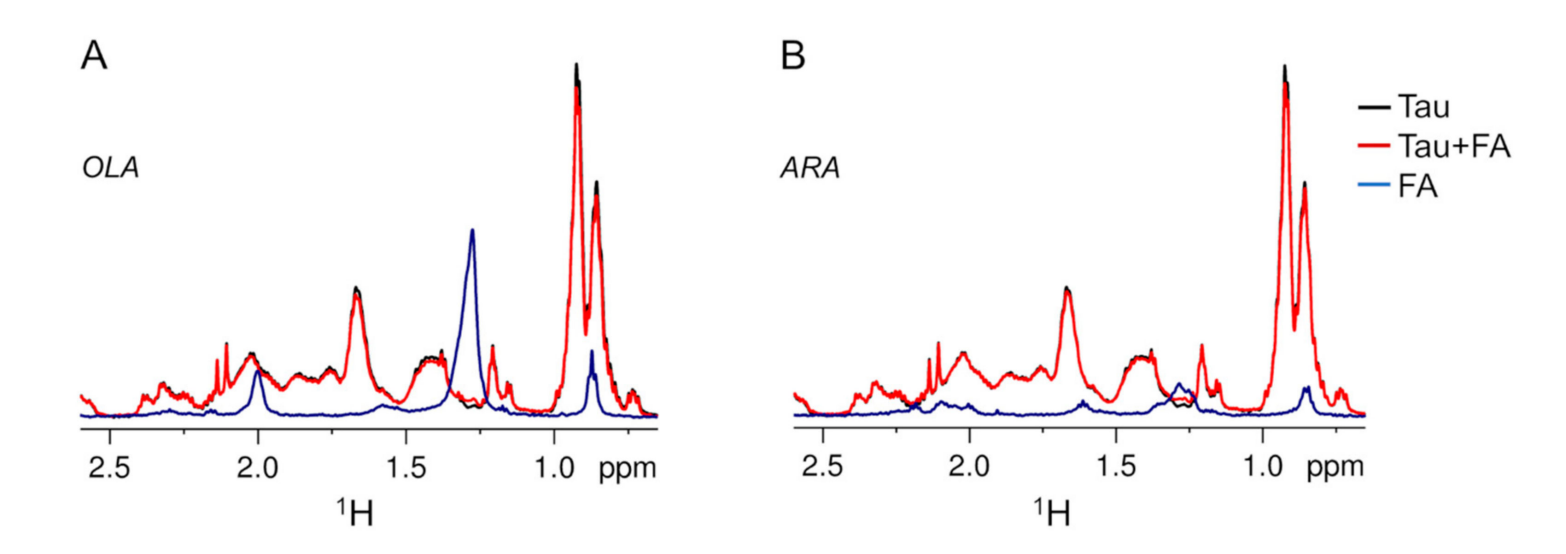
^1^H-NMR profiles of Tau^4RD^ and fatty acids. High-field portion of ^1^H-NMR spectra of 25 μM Tau^4RD^ (black), 75 μM fatty acid (FA, blue), and 25 μM Tau^4RD^ mixed with 75 μM FA (red). (**A**) Data obtained with FA = oleic acid (OLA). (**B**) Data obtained with FA = arachidonic acid (ARA). Measurements were performed at 37 °C.

**Figure 2:**
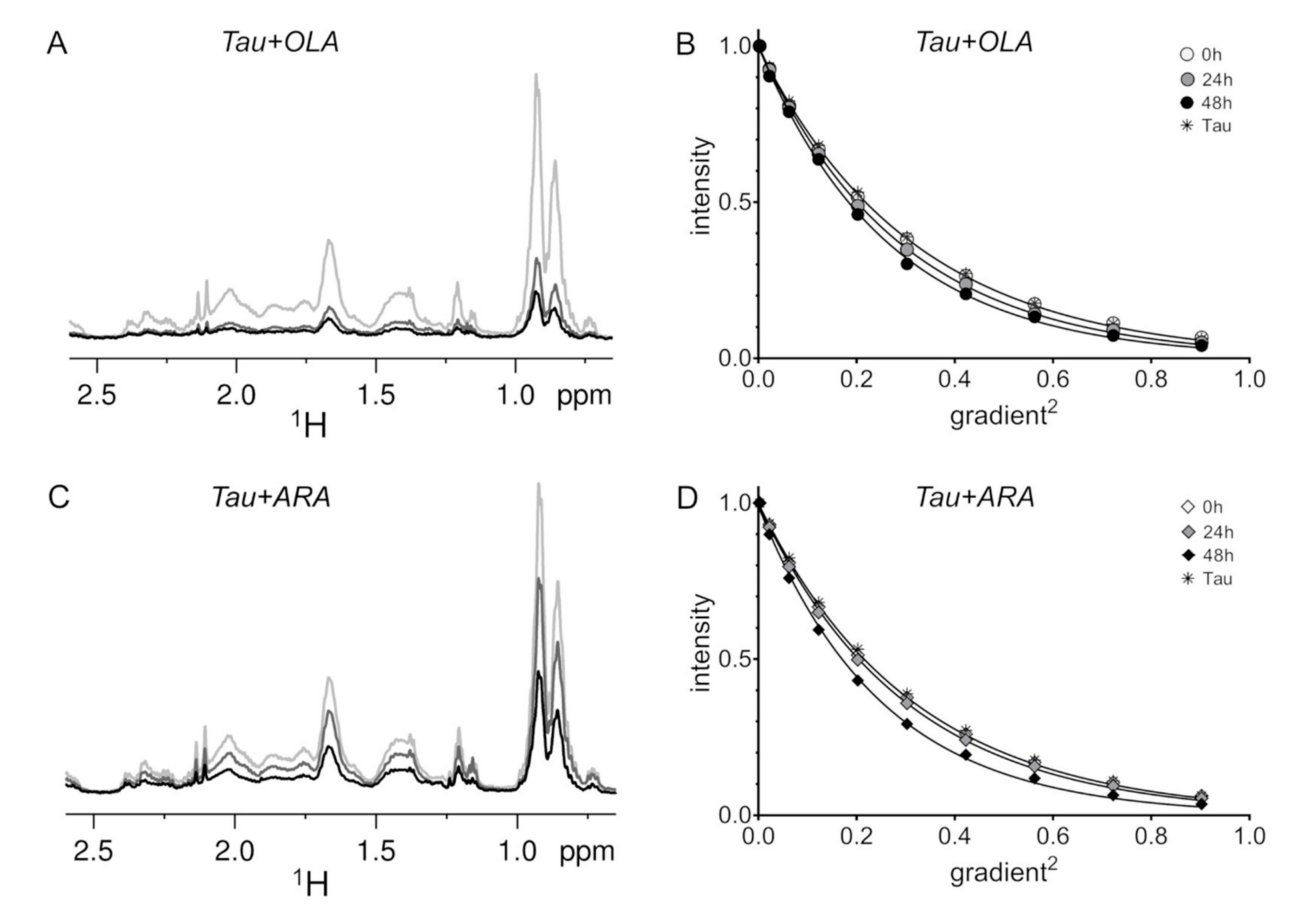
^1^H-NMR measurements of Tau^4RD^ monomer depletion and translational diffusion. High-field region of ^1^H-NMR spectra (**A**) and translational diffusion experiments (**B**) recorded on Tau^4RD^ in the presence of OLA. High-field region of ^1^H-NMR spectra (**C**) and translational diffusion experiments (**D**) recorded on Tau^4RD^ in the presence of ARA. Samples contained 25 μM Tau^4RD^ and 75 μM fatty acid. Spectra in (**A**,**C**) were collected on samples that were incubated for 0 h (gray), 24 h (dark gray), and 48 h (black). In (**B**,**D**), the normalized intensity of a selected signal is shown as a function of the square of the fraction of gradient used; errors in peak intensities were smaller than the size of the symbols, as estimated from duplicate experiments; samples were incubated for 0 h (empty circles/diamonds), 24 h (gray filled circles/diamonds), and 48 h (black filled circles/diamonds); asterisks denote data collected on Tau^4RD^ in the absence of fatty acid; lines are fitted exponential curves. Protein samples were aliquots taken from 100 μM protein solutions incubated with 300 μM fatty acid under magnetic stirring. Samples were kept at 37 °C during both incubation and measurement.

A preliminary investigation of the interaction between FAs and Tau^4RD^ was performed by acquiring simple 1D ^1^H-NMR spectra on protein/FA mixtures. All of the samples investigated in this work contained excess sodium dithionite to mimic the reducing environment of a neuronal cell body. The addition of a three-fold molar excess of FA to Tau^4RD^ produced minor perturbations of the protein ^1^H-NMR profile: virtually no chemical shift changes were observed and intensities were only slightly reduced (Figure 1) to an extent that was dependent on the ionic strength (Figure 1B and Figure S2). On the contrary, the signals of the FAs became undetectable and they did not add to the protein signals (Figure 1). Based on translational diffusion measurements, the observable protein ^1^H-NMR signals could be attributed to Tau^4RD^ molecules, which were freely diffusing in solution and were not part of larger aggregates (Figure 2B,D). Thus, a small portion of protein molecules appeared to interact with the bulk of FAs, determining a major change in lipid aggregation state and/or dynamics. It is noteworthy that the addition of Tau^4RD^ to a solution of ARA determined a reduction in sample turbidity measured at 350 nm (Figure S3A,C), likely due to a decreased size of assemblies present in the mixture compared to lipid-only solution. The effect was similar, although less pronounced with OLA (Figure S3A,C). For comparison purposes, we investigated the interaction of FAs with another intrinsically disordered amyloidogenic protein, α-synuclein (αS), which is known to associate with cellular lipids [38,41,42]. Indeed, the turbidity of FA solutions was significantly decreased in the presence of αS (Figure S3B,D), as observed previously [31], indicating that both intrinsically disordered proteins (IDPs) tend to disperse lipid aggregates to an extent that depends on the chemistry of the polypeptide chains.

**Figure 3:**
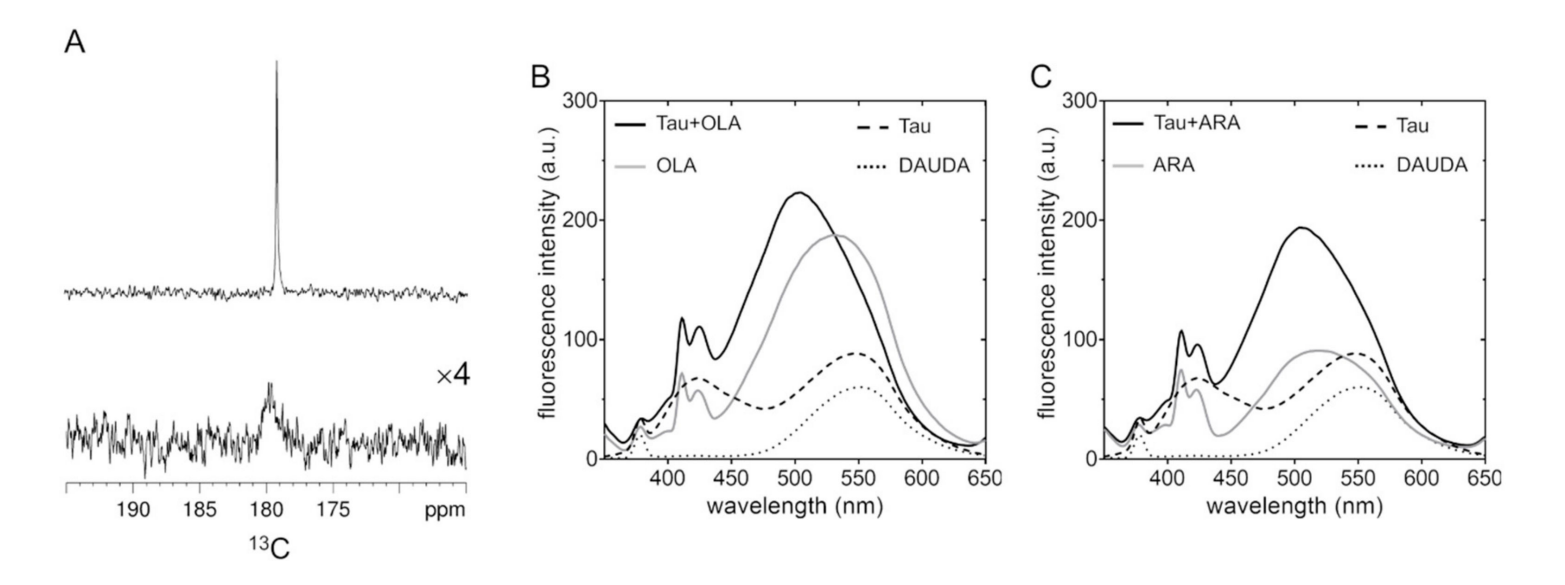
Perturbations of the chemical environment of fatty acids in the presence of Tau. (**A**) 1D ^13^C spectrum measured on 80 μM [^13^C_1_]OLA in the absence (top) and presence (bottom) of 20 μM Tau. The intensity of the bottom spectrum is increased four-fold compared to the top spectrum for better visualization. Measurements were performed at 37 °C. (**B**) DAUDA fluorescence spectra recorded on samples containing 300 μM OLA in the absence (gray solid line) or presence of 100 μM Tau^4RD^ (black solid line). (**C**) DAUDA fluorescence spectra recorded on samples containing 300 μM ARA in the absence (gray solid line) or presence of 100 μM Tau^4RD^ (black solid line). Spectra of control samples containing buffer (dotted line) or Tau4RD (dashed line) without FA are displayed in both (**B**,**C**). DAUDA was 1 μM in all samples.

To further examine the changes in the chemical environment experienced by the FAs, we recorded 1D ^13^C-NMR spectra of ^13^C_1_-enriched OLA, which allowed us to observe the FA without overlap from protein signals. The single sharp peak observed for [^13^C_1_]OLA alone in solution (Figure 3A) indicates that the carboxylates of OLA molecules experience a unique, homogeneous environment and exhibit fast rotational dynamics. After the addition of Tau^4RD^, the signal was considerably attenuated and broadened, suggesting that the protein perturbed the original aggregated state of OLA, determining changes in mobility and/or establishing heterogeneous non-covalent interactions.

To gain independent evidence of the Tau-mediated reorganization of FA assemblies, we performed fluorescence measurements in the presence of the environment-sensitive fluorescent fatty acid analogue DAUDA. In a solution of OLA (without protein), the DAUDA fluorescence emission peak exhibited a substantial increase in intensity and a blue-shift of the maximum emission wavelength (λ_max_ = 532 nm) from its position in the absence of FA (λ_max_ = 552 nm) (Figure 3B). In the case of ARA, the change in intensity was smaller and the shift of the peak was larger (λ_max_ = 517 nm) (Figure 3C), suggesting a different supramolecular organization of the FA aggregates (in the absence of protein) or a different mode of interaction with the fluorescent probe. In a solution of Tau^4RD^, the DAUDA fluorescence peak exhibited a modest increase in intensity and no change in wavelength compared to DAUDA alone. A second, unassigned peak appeared at shorter wavelengths. Interestingly, the fluorescence spectra of the Tau^4RD^/OLA and Tau^4RD^/ARA samples were remarkably similar both in intensity and maximum emission wavelength (λ_max_ = 504 and 505 nm for ARA and OLA, respectively). Thus, fluorescence spectra indicate that Tau^4RD^ perturbs the FA supramolecular organization and mediates the formation of aggregates with similar properties for both FAs.

### 2.3. Site-Resolved Investigation of Fatty Acid-Induced Perturbations of Tau^4RD^

**Figure 4:**
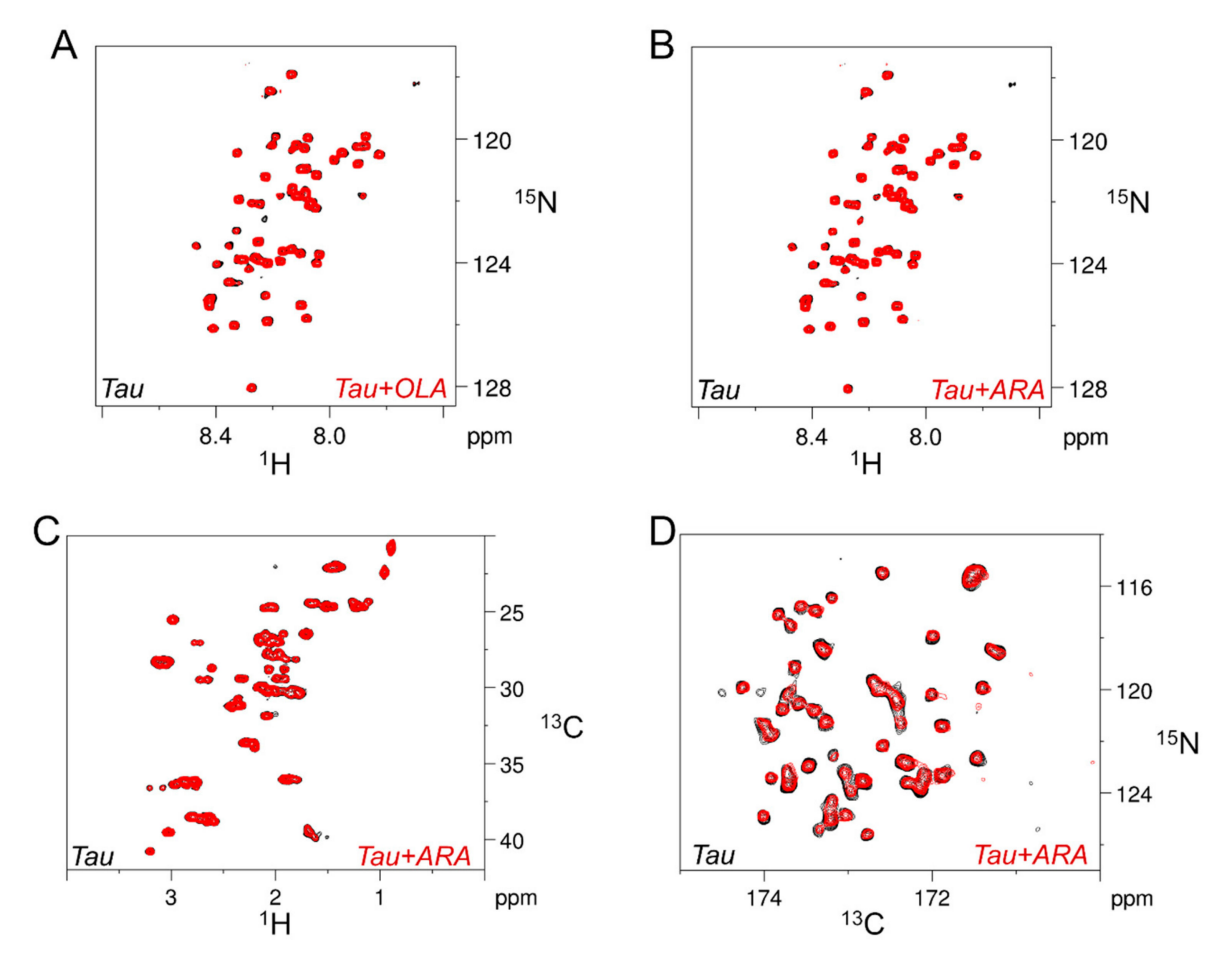
Fatty acid-induced NMR signal perturbations monitored at single residue/atomic resolution. (**A**, **B**) Overlaid, selected regions of HN-HSQC spectra recorded on [^15^N]Tau^4RD^ in the absence (black) or presence of OLA (**A**, red) or ARA (**B**, red); measurements were performed at 25 °C; protein concentration was 25 μM and fatty acid concentration was 75 μM. (**C**) Overlaid, selected regions of HC-HSQC spectra recorded on [^15^N,^13^C]Tau^4RD^ in the absence (black) or presence of ARA (red); the measurement temperature was 37 °C; protein concentration was 100 μM and fatty acid concentration was 50 μM. (**D**) Overlaid, selected regions of CON spectra recorded at 37 °C on samples as in panel C.

One-dimensional ^1^H-NMR profiles provided readily accessible information on Tau^4RD^/FA interactions, albeit at low resolution. To analyze the interactions in greater detail, we acquired a number of two-dimensional (2D) heteronuclear correlation spectra. The HN-HSQC spectrum of ^15^N-enriched Tau^4RD^ displays fairly well-resolved resonances from individual amino acid residues due to the significant dispersion of chemical shifts in the ^15^N frequency dimension and the narrow line shapes. After the addition of an excess of either OLA or ARA, the protein peak pattern appeared unmodified, with no signals exhibiting chemical shift changes nor residue-specific intensity variations (Figure 4A,B). It should be noted that the HN-HSQC spectra were acquired at 25 °C, a condition in which the sensitivity of the experiment was acceptable. At higher temperatures, the HN resonances were strongly attenuated due to fast amide proton exchange with the solvent, a major limitation of disordered polypeptides [43].

To explore whether an increase in temperature could facilitate the observation of FAs binding to specific protein sites, we performed alternative experiments based on non-exchangeable atoms. Both HC-HSQC and CON spectra were recorded at 37 °C on ^15^N,^13^C-enriched Tau^4RD^ (Figure 4C,D). The former experiment is very sensitive and displays signals from every H-C atom pair, although certain regions appear quite crowded. The latter is less sensitive but exhibits excellent single-residue resolution [44]. Careful comparison of the corresponding spectra acquired in the absence and presence of ARA revealed that no atom pair of the observable protein state was perturbed by the presence of FAs.

**Figure 5:**
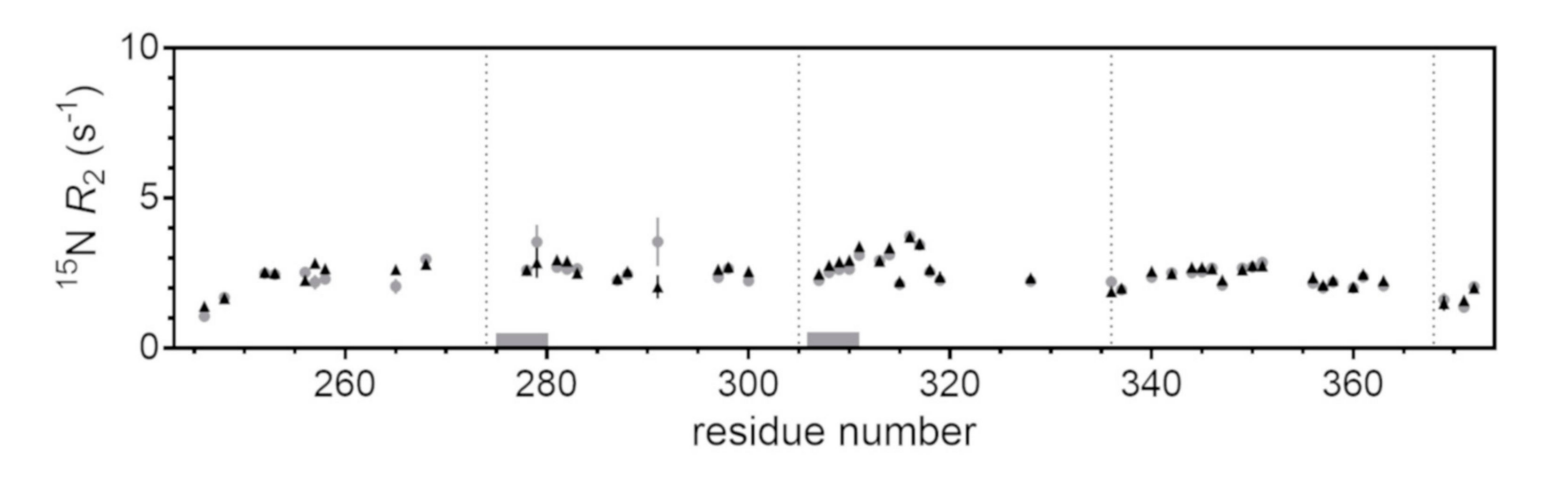
^15^N transverse relaxation rates. ^15^N-spin transverse relaxation rate values measured on 100 μM [^15^N]Tau^4RD^ in the absence (gray dots) and presence of 60 μM ARA (black triangles). Measurements were performed at 25 °C and a magnetic field of 14.1 T. Error bars are fitting errors. Dotted vertical lines indicate the position of separation between distinct repeats. Gray horizontal bars indicate the position of the hexapeptide motifs. The amino acid residue numbering is based on full-length human Tau.

In principle, the formation of transient, small protein/FA complexes may escape direct detection by all of the above NMR methods, however, additional experiments may reveal the presence of low-populated states and dynamic association equilibria [45]. For this reason, we carried out ^15^N-spin transverse relaxation rate, ^15^N-*R*_2_, measurements, which are sensitive to local motional properties [46]. ^15^N-*R*_2_ values were relatively homogeneous throughout the polypeptide sequence (Figure 5), consistent with the disordered nature of Tau^4RD^, and appeared essentially unperturbed after the addition of ARA, definitively supporting the conclusion that the observable protein state was not perturbed by FA molecules.

Two-dimensional ^13^C spectra and ^15^N-spin transverse relaxation data were collected with excess protein, at variance with most other experiments in this work, in order to ensure adequate signal/noise by mitigating the signal attenuation caused by the presence of excess FA. Both types of experiments aimed at detecting intermolecular interactions and exchange based on the perturbations of the free-state signals, as opposed to other experiments performed to directly observe conformational transitions and aggregation.

### 2.4. Time-Dependent Tau^4RD^ Monomer Depletion and Translational Diffusion

Tau^4RD^ is known to undergo self-association in the presence of aggregation inducers. To monitor the progress of protein aggregation, ^1^H-NMR spectra were recorded on Tau^4RD^/FA samples immediately after mixing and after 24 h or 48 h of incubation under reducing conditions. We note that no major FA oxidation products were detected by the NMR analysis of FA samples incubated for one or two days. In the presence of OLA, the spectrum intensity decreased by 67% after 24 h and 78% after 48h (Figure 2A), indicating that the population of free protein was reduced due to formation of protein-containing aggregates. Unexpectedly, the translational diffusion of the species observed in the spectrum did not decrease, but instead was slightly faster (~10%) compared to that measured on the freshly prepared mixture (Figure 2B). This result can likely be attributed to the structural compaction induced by the formation of intramolecular disulfide bridges [47] upon the progressive decrease of DTT reducing activity. In the case of ARA, the protein spectrum displayed reduced intensity after 24 h (30% reduction) and after 48 h (60%) (Figure 2C). Again, it was remarkable to observe that the protein diffusion coefficient became larger (~20% at 48 h) after prolonged incubation with the FA (Figure 2D). Thus, the timescale of monomer-aggregate exchange was larger than the pulse separation delay (200 ms) of translational diffusion measurements.

### 2.5. FA-Induced Protein Secondary Structure Perturbations

**Figure 6:**
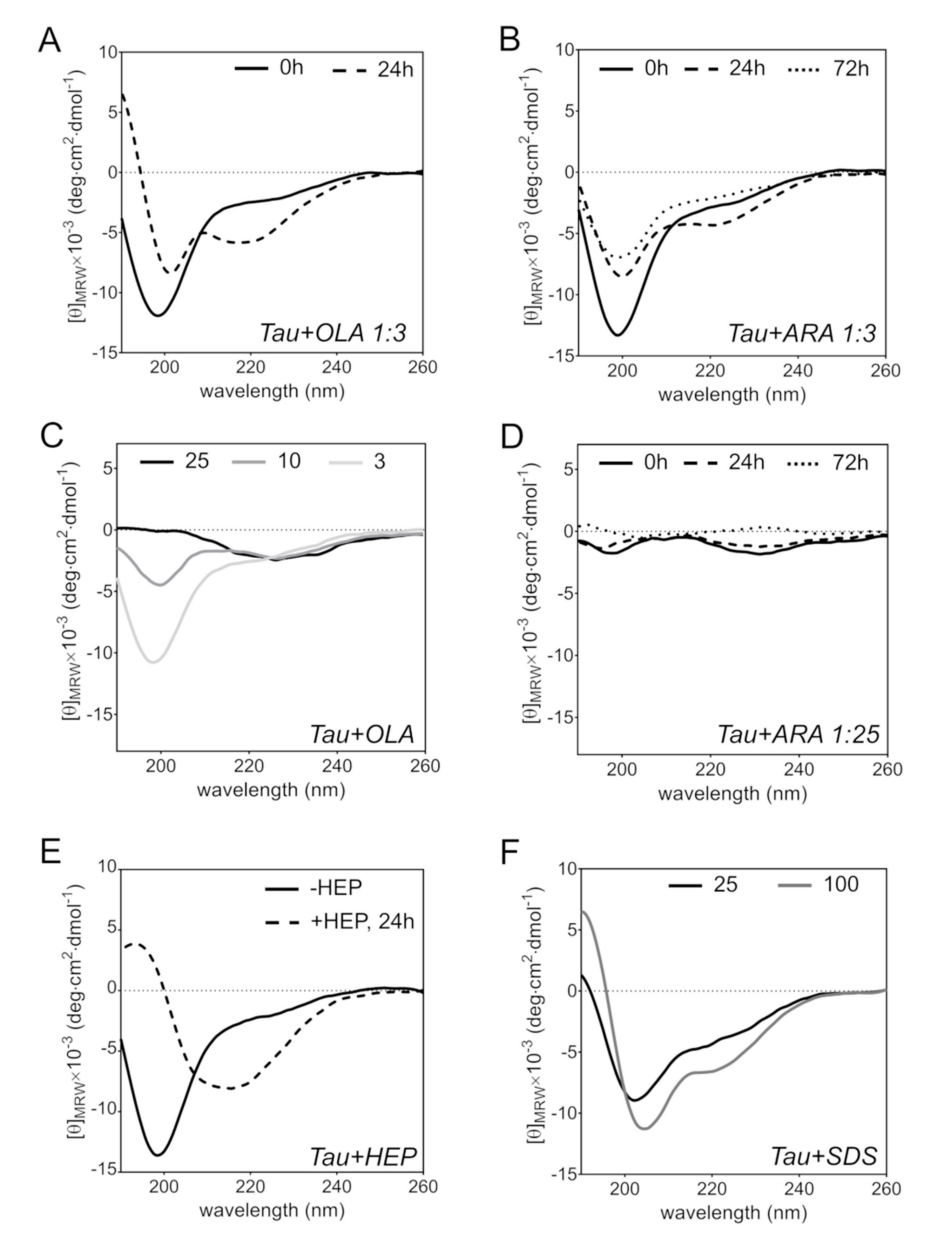
Far-UV circular dichroism spectroscopy. (**A**) Tau^4RD^ in the presence of OLA immediately after mixing (solid line) and after 24 h of incubation (dashed) at 37 °C under magnetic stirring, at a molar ratio Tau^4RD^:OLA = 1:3. (**B**) Tau^4RD^ in the presence of ARA immediately after mixing (solid line) and after 24 h (dashed) or 72 h (dotted) of incubation at 37 °C under magnetic stirring, Tau^4RD^:ARA = 1:3. (**C**) Tau^4RD^ in the presence of OLA immediately after mixing, Tau^4RD^:OLA = 1:3 (light gray), 1:10 (gray), 1:25 (black). (**D**) Same as B, with Tau^4RD^:ARA = 1:25. (**E**) Tau^4RD^ (solid) and Tau^4RD^ mixed with heparin (HEP), after 24 h incubation (dashed), Tau^4RD^:HEP = 4:1. (**F**) Tau^4RD^ (solid) and Tau^4RD^ immediately after mixing with SDS, Tau^4RD^:SDS = 1:25 (black), 1:100 (gray). [θ]_MRW_ is the mean residue molar ellipticity.

**Figure 7:**
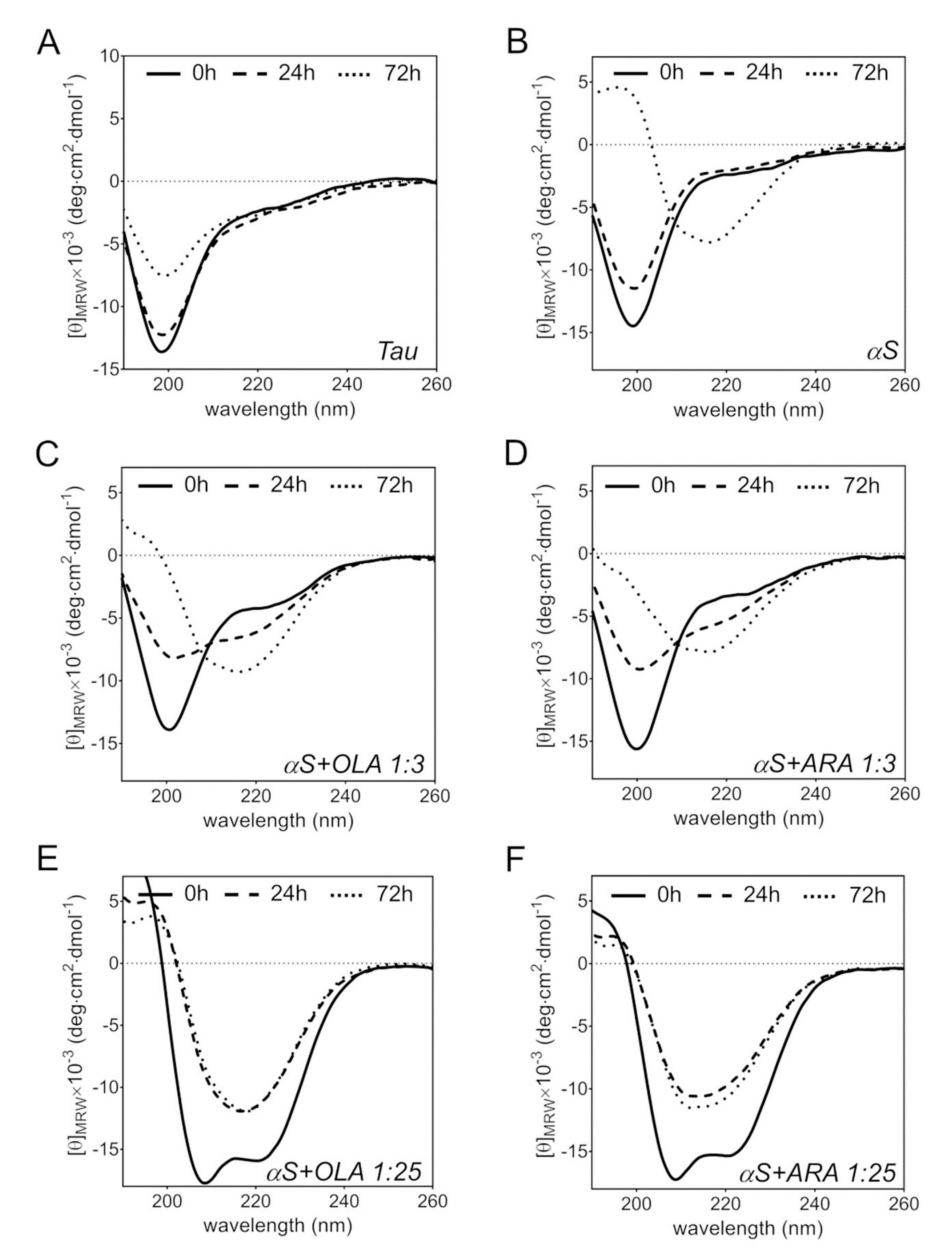
Far-UV circular dichroism spectroscopy. (**A**) Tau^4RD^ freshly prepared (solid line) and after 24 h (dashed) or 72 h of incubation (dotted) at 37 °C under magnetic stirring. (**B**) αS freshly prepared (solid line) and after 24 h (dashed) or 72 h of incubation (dotted) at 37 °C under magnetic stirring. (**C**) αS in the presence of OLA immediately after mixing (solid line) and after 24 h (dashed) or 72 h (dotted) of incubation at 37 °C under magnetic stirring, αS:OLA = 1:3. (**D**) αS in the presence of ARA immediately after mixing (solid line) and after 24 h (dashed) or 72 h (dotted) of incubation at 37 °C under magnetic stirring, αS:ARA = 1:3. (**E**) Same as C, αS:OLA = 1:25. (**F**) Same as D, αS:ARA = 1:25. [θ]_MRW_ is the mean residue molar ellipticity.

We used circular dichroism (CD) spectroscopy to gain insight into changes in the secondary structure of Tau^4RD^, induced by the presence of FAs. The far-UV CD spectrum of Tau^4RD^ is characteristic of a largely disordered protein, featuring a deep minimum centered at 198 nm and the absence of strong negative signals above 205 nm (Figure 6E, Figure 7A). On the addition of a three-fold molar excess of OLA, the protein spectrum remained unperturbed (Figure 6A), however, after incubation for 24 h, significant changes were observed. The lineshape was characterized by two minima, at 201 and 218 nm, and positive ellipticity below 195 nm, which can be attributed to a mixture of protein molecules in disordered states and in partially ordered conformations, however, with no uniquely defined secondary structure elements. In the presence of a larger excess of OLA, the strong negative peak at 198 nm became significantly attenuated (Figure 6C), suggesting the progressive depletion of free disordered protein molecules.

In analogy with OLA, the addition of ARA did not immediately perturb the CD spectrum of Tau^4RD^, but changes were observed after a longer incubation. The spectrum recorded after 24 h of incubation exhibited two minima, centered at about 200 and 221 nm (Figure 6B), which became less pronounced after 72 h. In the presence of a 25-fold excess of ARA, the negative ellipticity peak of the disordered state was essentially canceled and no other major peak appeared during the course of the incubation (Figure 6D). Thus, under these conditions, the protein partitioned, to a greater extent, into aggregates.

To better understand the structural perturbations induced by FAs, experiments were also performed with the anionic aggregation-inducer heparin and the amphiphilic detergent SDS. The transition of Tau^4RD^ from the disordered state to a β-sheet structure after incubation with heparin for 24 h was clearly evident from the CD spectrum (Figure 6E), in agreement with previous studies [48]. By contrast, the addition of SDS produced the quantitative conversion to an α-helical structure (Figure 6F), as reported previously [49]. The spectrum lineshapes of Tau^4RD^ in the presence of FAs were clearly distinct from those observed in these two limiting cases of mostly β or α secondary structure conformations, supporting the view of mixed-nature secondary structure content and partly disordered states.

For comparison purposes, we also performed measurements with αS. The latter can easily form amyloid fibrils in vitro without the addition of inducers, and the corresponding CD spectrum, recorded after 72 h of incubation, was consistent with the presence of a β-sheet structure (Figure 7B). After the addition of a three-fold molar excess of OLA or ARA, the characteristic strong negative peak, centered at 201 nm, remained unperturbed, indicating that partitioning into FA assemblies did not make a significant contribution to the spectral lineshape (Figure 7C,D). However, after 24 h, the peak was considerably reduced and negative ellipticity increased at wavelengths close to 217 nm. The latter band became progressively stronger with increasing incubation time, while the peak of the disordered state disappeared (Figure 7C,D). Interestingly, upon the exposure of αS to a 25-fold molar excess of FA, the protein turned immediately into an α-helical state (Figure 7E,F) but transitioned rapidly to a β-sheet after 24 h, in agreement with the results of Klenerman and coworkers for the ARA-induced structural rearrangement of αS [50]. Taken together, the data indicate that OLA and ARA had the same effects on the conformational properties of αS.

### 2.6. FA-Induced Fibril Formation

**Figure 8:**
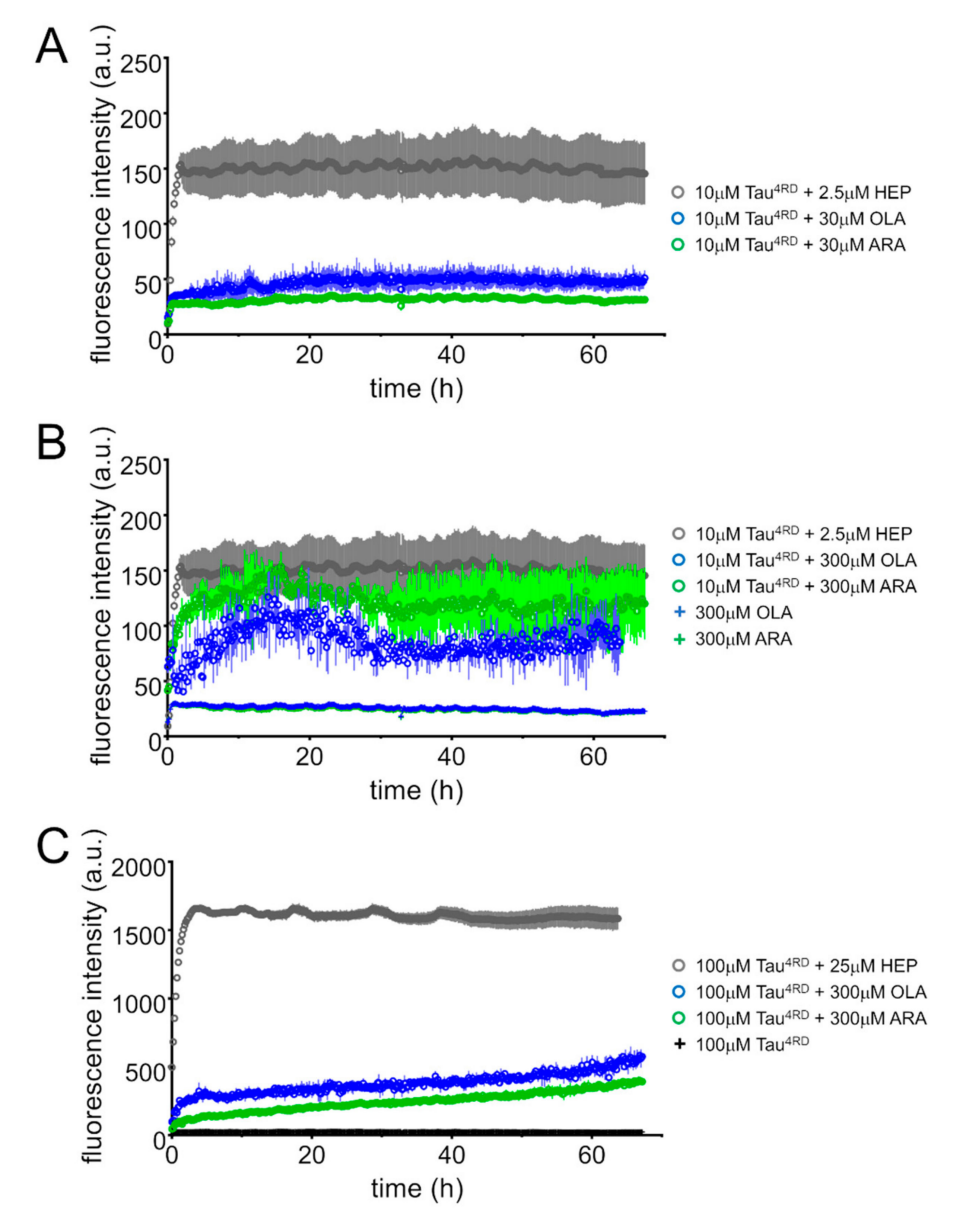
Thioflavin T (ThT) fluorescence assays. ThT fluorescence intensity was monitored over time on samples containing: (**A**) 10 μM Tau^4RD^ and 30 μM fatty acid (FA); (**B**) 10 μM Tau^4RD^ and 300 μM FA; (**C**) 100 μM Tau^4RD^ and 300 μM FA. Protein and FAs were dissolved in phosphate buffer, pH 6.8, also containing excess dithiothreitol (DTT). Samples were incubated at 30 °C under intermittent agitation. Control measurements were performed on protein (**C**) and FAs (**B**). Aggregation kinetic curves for Tau^4RD^ in the presence of heparin (HEP) are displayed for comparison (grey circles). The displayed data are the mean ± SD of measurements performed in quadruplicate (no error bars are shown for control samples).

After having ascertained that both OLA and ARA promote the progressive depletion of free soluble Tau^4RD^ molecules, its association to lipid aggregates and the transition towards conformations with mixed-type secondary structures, we set out to explore the formation of fibrils. We used the thioflavin-T (ThT) fluorescence assay to follow protein polymerization over time. The ThT fluorescence intensity of samples containing 10 μM protein and 30 μM FAs did not increase significantly during the incubation period (Figure 8A), as opposed to the rapid increase observed when the protein was incubated with heparin. When the same concentration of protein was presented with a large excess of FAs, the fluorescence rapidly increased, reaching a maximum, after ca. 12 h of incubation, of similar intensity (in the case of ARA) to that observed with heparin (Figure 8B). The fluorescence decreased during the following incubation period until it reached a constant value after about 30 h. The observed trend suggested the formation of ThT-positive protein polymers. The ThT fluorescence curves measured on more concentrated Tau^4RD^ samples displayed a rapid increase during the first few hours of incubation, followed by a slower but constant increment during the following hours (Figure 8C). Although the fluorescence change was significant, the maximum observed intensity remained much lower than that measured upon incubation with heparin, suggesting that ThT-positive polymers were produced in smaller quantities or that the ThT affinity was reduced in the presence of FAs.

**Figure 9:**
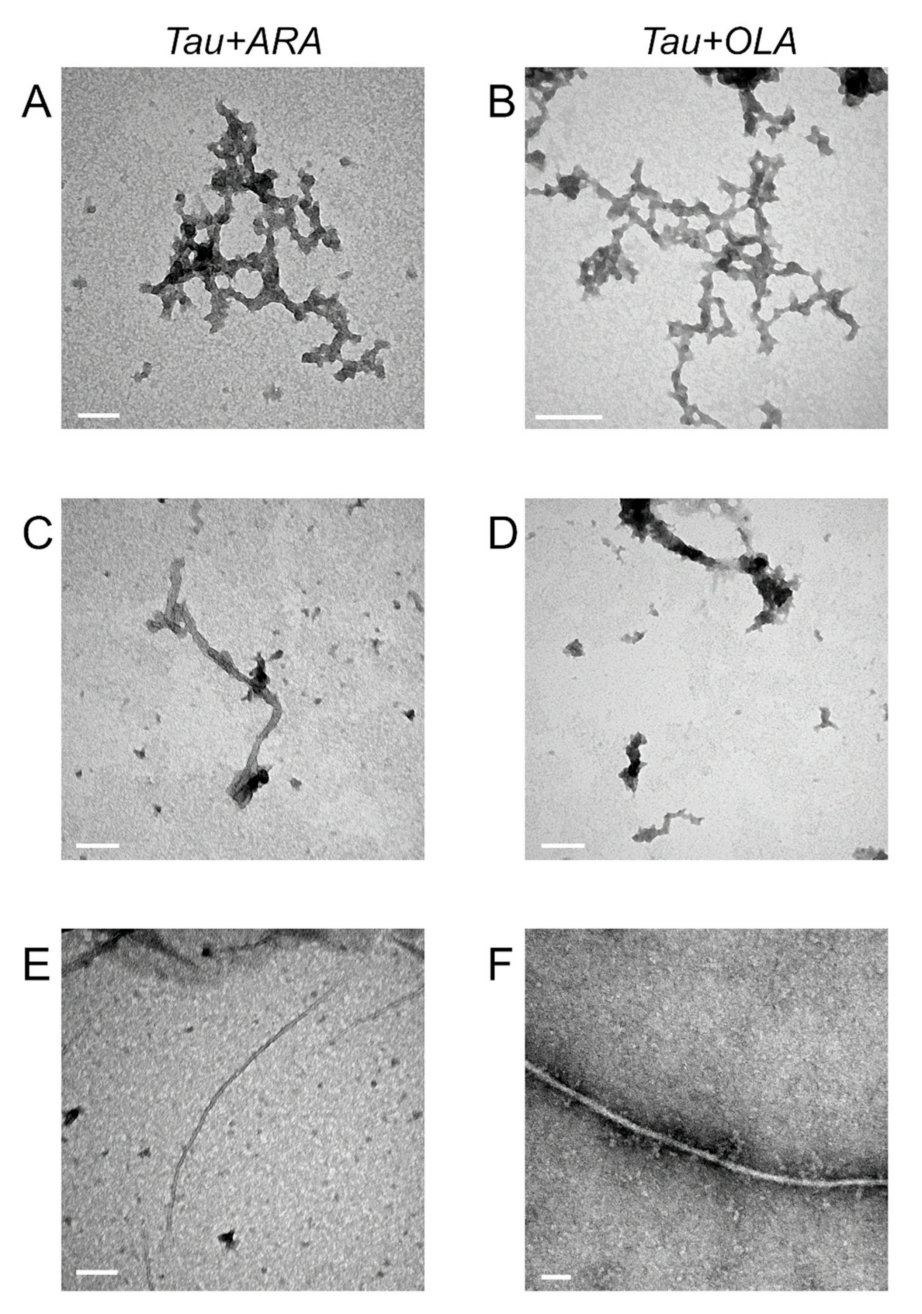
TEM analysis of protein/FA aggregates. TEM micrographs were taken on Tau^4RD^/FA samples incubated for 24 h at 30 °C under intermittent shaking. Samples contained: (**A**,**B**) 10 μM protein and 30 μM FA; (**C**,**D**) 10 μM protein and 300 μM FA; (**E**,**F**) 100 μM protein and 300 μM FA. Scale bars are 100 nm.

We used TEM to verify the presence of protein fibrillar aggregates produced during incubation with FAs. The micrographs obtained on samples of 10 μM protein/30 μM FA displayed the presence of amorphous assemblies (Figure 9A,B, Figures S5 and S6), similar to those found in the absence of protein (Figure S4). The images of samples obtained from 10 μM protein/300 μM FA mixtures revealed the presence of a few poorly resolved filamentous structures, covered by lipid aggregates (Figure 9C,D, Figures S5 and S6). Well-resolved filaments were instead observed for samples prepared from 100 μM protein/300 μM FA (Figure 9E,F, Figures S5 and S6), consistent with the larger increase in ThT fluorescence observed during incubation. Tau^4RD^ did not form fibrils in the absence of inducers (AFM data, not shown). The slightly bent, unbranched, and untwisted single filaments obtained with ARA were thinner (7 ± 0.8 nm width, *n* = 16) than those obtained with OLA (16 ± 3 nm, *n* = 25).

## 3. Discussion

The interaction between Tau and lipids has been proposed to play an important role in the function and dysfunction of this protein [25,36,49,51], however, much of the reciprocal influence between these molecules remains poorly understood, particularly as far as FAs are concerned. In a bid to elucidate the association of Tau with unsaturated FAs at the sub-molecular level, we carried out a variety of solution NMR experiments in combination with CD and fluorescence measurements.

Solutions of both ARA and OLA in the concentrations used for NMR experiments (75–500 μM) contained a major fraction of self-assembled lipid molecules, as determined from translational diffusion measurements, while unassociated ARA molecules were also detected at the lowest concentration. After mixing Tau^4RD^ with the FAs, the NMR signals of the latter vanished and the ^1^H-NMR profile was almost coincident with the spectrum of the protein (Figure 1). Thus, in the presence of Tau^4RD^, the lipid assembly transitioned to a new state characterized by slower molecular rotational diffusion and/or increased supramolecular heterogeneity, both situations consistent with signal broadening and attenuation. This conclusion was further supported by the observation of a dramatic attenuation and broadening of the ^13^C-NMR signal of [^13^C_1_]OLA in the presence of Tau^4RD^, compared to the corresponding signal in a protein-free solution (Figure 3A).

Interestingly, the fluorescence emission band of the fluorescent FA-analogue DAUDA exhibited a stronger intensity and lower maximum emission wavelength in Tau^4RD^/FA mixtures than in FA-only solutions (Figure 3B,C). The fluorescence data indicated that the fluorophore was exposed to a more hydrophobic environment in the co-aggregates than in the lipid assemblies, suggesting that the protein-induced reorganization of the lipid aggregates allowed the FA-analogue to better approach the hydrophobic hydrocarbon chains. The similarity of the fluorescence spectra measured on the protein/ARA and protein/OLA systems suggests that the two types of co-aggregates shared the same supramolecular organization, which was therefore independent from the degree of unsaturation of the FAs.

Previous studies [52] indicated that full-length Tau reduced the critical micellar concentration of a number of FAs and detergents, and our turbidity measurements revealed an apparent reduction in the size of lipid assemblies in the presence of Tau^4RD^. An analogous behavior was observed with another prototypical amyloidogenic protein, αS. However, the view of a protein-induced depression of the critical micellar concentration might be too simplistic: the physical states of long-chain FAs are rather complex and heterogeneous mixtures of distinct aggregate species, including oil droplets and lamellar phases, may coexist at neutral pH [39]. It is therefore not straightforward to predict what kind of reorganization occurs in the presence of Tau^4RD^. Previous studies with model lipid vesicles and structured membranes established that a three-repeat domain of Tau adsorbed to lipid bilayers without deep penetration into the membrane [53], however, both Tau^4RD^ and full-length Tau induced morphological changes in phospholipid vesicles [36] or disruptions to membrane lipid packing and structural integrity [54]. Therefore, it appears that a polyelectrolyte, such as Tau^4RD^, can strongly perturb both phospholipids, as well as FA supramolecular organization, presumably through a combination of ion-pairing attractive forces and nonpolar interactions involving the protein’s hydrophobic motifs.

The presence of Tau^4RD^ in protein-lipid co-aggregates was initially inferred from the protein-induced perturbations to FAs and from a small general reduction of the intensity of protein NMR signals. Using a variety of heteronuclear correlation spectra, we were able to monitor distinct atom pairs (HN, CH, NCO) of Tau^4RD^ after the addition of FAs (Figure 4). None of these reporter groups was found to experience chemical shift perturbations nor changes in relaxation properties or translational diffusion (Figure 2, Figure 4 and Figure 5), indicating that the observable signals belonged to free protein molecules. The absence of detectable changes in any of the NMR observables indicates that the lipid-bound state was too sparsely populated or that the free and bound states were separated by a high energy barrier at temperatures of 25 °C or 37 °C, i.e., the dissociation kinetics were very slow on the experimental timescale. The negligible changes in the far-UV CD spectra of Tau^4RD^ recorded shortly after mixing with FAs (1:3 molar ratio) (Figure 6) support the view that a small fraction of protein molecules was involved in the initial co-aggregate formation. Only after the addition of larger amounts of FAs did major perturbations of the CD bands become visible, indicating that a substantial portion of protein molecules was interacting with lipids. Thus, the assemblies must have been characterized by a high lipid/protein molar ratio, in agreement with a central role of ion–ion interactions between the abundant positive charges of the protein and the single anionic head group of an FA molecule. The observed behavior was unique to the studied long-chain FAs: indeed, the addition of the detergent SDS induced the rapid transition of disordered Tau^4RD^ to an α-helix-containing conformational state and the binding of a single-cysteine mutant of Tau^4RD^ to lipid micelles was earlier shown to induce the formation of short amphipathic α-helical structures [49].

The initially formed aggregates were not stable but evolved over time: upon prolonged exposure to FAs, the fraction of unbound Tau^4RD^ species decreased, as indicated by the attenuation of the ^1^H-NMR spectral envelope after 24 h and 48 h (Figure 2). Despite the major involvement of protein molecules in aggregate formation, no perturbation of NMR observables (besides global signal attenuation) was detected and the molecular translational diffusion of the detectable species did not increase as one would have expected for a system in rapid exchange with larger aggregates. Therefore, the bound state was kinetically separated from the free state. The time-dependent evolution of the system was also particularly evident from CD spectra (Figure 6): in the presence of either OLA or ARA in three-fold molar excess, the characteristic band of the disordered conformation became less intense, while a second band centered around ~220 nm appeared, indicating an increased level of secondary structure.

The quantitative interpretation of the CD spectra of disordered polypeptides is not yet fully established [55] and the accurate analysis of the spectra of protein samples containing diverse aggregates may be further hampered by scattering or solvent shift effects and sample heterogeneity [56]. For this reason, instead of applying deconvolution tools, we preferred to perform phenomenological comparative analyses of spectra from samples of different protein/FA compositions. The exposure of Tau^4RD^ to heparin for a few hours induced a quantitative transition towards a conformational state with a clear β-sheet signature. A similar signature characterized an αS sample after 72 h of incubation without any cofactor or in the presence of FAs in either slight or large excess (Figure S3). Thus, the two similarly sized, but chemically distinct, polypeptides, Tau^4RD^ and αS, experienced quite distinct conformational transitions during prolonged exposure with OLA and ARA. Although Tau^4RD^ is capable of adopting α-helix-rich conformations when interacting with SDS or phospholipid membranes, this was not the case with FAs. Over time, αS converts into a β-rich conformation, while the conformations of Tau^4RD^ appear structurally heterogeneous with only the partial formation of secondary structure. The absence of unique secondary structure signatures for Tau^4RD^ in the presence of large excess of OLA or ARA suggests that the protein samples a complex conformational space and/or the molecules are distributed non-uniformly in the sample, being concentrated within co-aggregate particles (resulting in absorption flattening).

Our aggregation kinetic experiments and TEM measurements revealed that ARA acts as an aggregation inducer of Tau^4RD^, albeit less potent than heparin. Since earlier studies demonstrated this effect on diverse constructs of Tau, including full-length Tau, 0N4R, 1N4R, and 2N4R [25,28,57], together these findings allow the confirmation of the central role of the microtubule binding domain in the ARA-induced self-aggregation of Tau. Here, we further showed that, despite differences in the properties of lipid states, distinct long-chain unsaturated FAs, exemplified by ARA and OLA, appear to reorganize into similar supramolecular structures and to induce the analogous conformational rearrangements and aggregation of Tau in reducing conditions. With the exception of a slightly different filament width, OLA^−^ and ARA-induced Tau^4RD^ filaments appeared morphologically similar, and comparable to those of full-length Tau assembled with ARA [25]. PHFs, analogous to those found in AD brains [17], were also observed upon the exposure of full-length Tau to ARA for long periods [57]. Thus, single filaments may represent precursors of PHFs. Recent cryo-electron microscopy developments, which have begun to shed light onto the diversity of the filamentous assemblies of Tau with unprecedented resolution [18,58], could clarify whether FA-induced filaments morphologically resemble those found associated with distinct tauopathies.

The influence of OLA and ARA on the secondary structure formation and aggregation of Tau is markedly different from that exerted by sulfated glycosaminoglycans, phospholipids, and detergents, supporting the view that different classes of molecules drive the polypeptide along alternative pathways towards the formation of insoluble fibrillar aggregates.

We note that physiological concentrations of individual FAs are generally in the micromolar range, although with large local and temporal variability, but they can rise up to 300 μM during pathology [29,59]. Thus, the conditions used in this work are generally above physiological levels, but they are well suited to appreciate the behavior of Tau in the presence of FAs and the quantitative conformational changes which would otherwise require unrealistic experimental times or would escape detection due to low abundance. Learning about conformational transitions in the used conditions serves as a basis for a better understanding of putative interactions occurring in the brain.

## 4. Materials and Methods

### 4.1. Reagents

Heparin (H3393), dithiothreitol, thioflavin-T (T3516), sodium oleate (O7501), sodium arachidonate (SML1395), 11-(Dansylamino)undecanoic acid (DAUDA, 39235), and protease inhibitors, were purchased from Sigma Aldrich (St Louis, MO, USA).

### 4.2. Recombinant Protein Expression and Purification

Recombinant Tau^4RD^, corresponding to the 244–372 sequence of human Tau-441, was expressed and purified as described previously [60]. Briefly, the protein was expressed in BL21 (DE3) cells grown in LB medium at 37 °C for 5 h, with 0.5 mM IPTG. Protein purification was achieved by thermal treatment of the soluble bacterial extract (80–100 °C) and SP-ion exchange chromatography. To label Tau^4RD^ with ^15^N, or ^15^N and ^13^C stable isotopes, the *Escherichia coli* culture was grown in M9 minimal medium supplemented with ^15^NH_4_Cl (1 g/L), or ^15^NH_4_Cl (1 g/L) and ^13^C glucose (4 g/L). Purified Tau^4RD^ products were dialyzed in the final buffer with the addition of 2 mM DTT.

Recombinant human αS was produced in *E. coli* BL21 (DE3) cells and purified as reported [61,62].

### 4.3. Fatty Acid Samples Preparation

Sodium oleate, OLA, was dissolved in 500 µL of mQ H_2_O to a final concentration of 100 mM. Subsequently, the solution was aliquoted and stored at room temperature until needed.

Sodium arachidonate, ARA, was prepared by adding H_2_O (fluxed with nitrogen) directly to the vial obtained from the manufacturer to a concentration of 15 mM [63]. The solution was aliquoted in Wheaton glass serum bottles (Z113948) in 200–500 µL and clamped under nitrogen in a glovebox. Aliquots were stored at −20 °C until needed.

### 4.4. NMR Spectroscopy

NMR experiments were acquired on a Bruker Avance III spectrometer (Bruker, Karlsruhe, Germany), operating at 600.13 MHz ^1^H Larmor frequency, equipped with a triple resonance TCI cryogenic probe. NMR data were processed with Topspin 4.0.6 (Bruker) and analyzed with the software NMRFAM-SPARKY [64].

All protein samples were centrifuged at 10,000× *g* for 10 min to remove any insoluble particles prior to NMR measurement and the protein concentration was assessed by measuring absorbance at 280 nm. The typical Tau^4RD^ concentration was 25–100 µM. The FA concentration was 75–300 µM. The samples were prepared in 10 mM potassium phosphate buffer at pH 6.8, also containing 1 mM DTT, protease inhibitors with EDTA, 0.02% NaN_3_ (Sigma), and 8% ^2^H_2_O.

One-dimensional ^1^H-NMR experiments were acquired at 37 °C, with a standard pulse sequence incorporating the excitation sculpting water suppression scheme. A total of 16–64 transients were acquired over a spectral width of 9615 Hz, using a recycle delay of 4 s. The spectra were processed by applying an exponential window function prior to the Fourier transformation. Protein samples were aliquots taken from a concentrated solution (100 µM protein solution mixed with 300 µM FA) and diluted to a final concentration of 25 µM protein and 75 µM FA.

^1^H-^15^N heteronuclear single quantum coherence (HSQC) spectra were acquired at 25 °C and were conducted by acquiring 256 complex points in the t_1_ dimension and 2048 in the t_2_ dimension. A total number of 8–16 transients were acquired for each spectrum with an interscan delay of 1.2 s. Standard sequence schemes with pulsed field gradients were used to achieve suppression of the solvent signal and the cancellation of spectral artifacts. Protein samples were prepared as for 1D experiments.

Pulsed field gradient NMR diffusion experiments were performed at 37 °C, using the standard Bruker *ledbpgp2s1dwsse* pulse sequence, which implements the stimulated echo and longitudinal eddy current delay schemes, as well as bipolar gradient pulses for diffusion. Water signal suppression was achieved by pre-saturation and Watergate. The gradient was set to values in the range of 5–95% of the maximum gradient strength. The experiment was conducted with diffusion and gradient times of 200 ms and 4 ms. Data were analyzed by integrating resonances of the amide envelope region. Samples were prepared by incubating 100 µM protein solution with 300 µM FA under magnetic stirring at 37 °C. At various time intervals, aliquots were taken and subsequently diluted for NMR measurements.

^15^N-spin transverse relaxation rate (^15^N-*R*_2_) measurements were carried out at 25 °C, in gradient-selected sensitivity enhanced mode and in an interleaved fashion acquiring 2048 (^1^H) × 256 (^15^N) complex data points for each relaxation delay over spectral widths of 12 (^1^H) and 26 (^15^N) ppm. Water signal suppression was obtained with optimized flip-back pulses. The recycle delay was set to 1.5 s and *T*_2_ (=*R*_2_^−1^) relaxation delays were 0.01696 s (×2), 0.1696 s, 0.2035 s (×2), and 0.2374 s (×3). Protein samples were prepared with 100 µM [^15^N]Tau^4RD^ mixed with 60 µM ARA. Sequence-specific assignments of NH resonances were obtained by visual transfer from published assignments (BMRB Entry 19,253 and in part from entry 17945) using a set of HSQC spectra recorded in progressively different buffer conditions and temperature to best match the distribution of peaks in the references [49,65]. Signals of Cys322 and nearby residues were not assigned because in the reference construct the cysteine was mutated.

Constant time (CT) ^1^H-^13^C HSQC spectra and CON spectra were acquired at 37 °C on samples containing 100 µM [^15^N,^13^C]Tau^4RD^ mixed with 50 µM FA. For CT-HSQC, acquisition parameters were optimized for the observation of the aliphatic resonances and spectra were recorded in a matrix of 2048 × 676 complex points on spectral windows of 16 ppm and 70 ppm in the ^1^H and ^13^C frequency dimensions, respectively, with the carbon carrier placed at 40 ppm, the recycle delay was set to 2 s, and the constant time evolution period was 53.2 ms. Carbon-detect CON spectra were recorded with IPAP virtual decoupling [66], 896 × 256 complex data points, 40 ppm (^13^C) × 48 ppm (^15^N) spectral windows, 64 scans, and a recycle delay of 2.5 s.

One-dimensional ^13^C-NMR experiments were performed at 37 °C to examine the carboxyl signals of [^13^C_1_]OLA, as described in previous work [67,68,69]. Samples contained 80 µM [^13^C_1_]OLA in the absence or presence of 20 µM Tau. Experiments were acquired with inverse-gated decoupling using a 30° flip angle for the carbon pulse. A pulse interval of 1.5 s, spectral width of 60 ppm centered at 170 ppm, and 16,384 time domain points were used for 4096 spectral accumulations. A line-broadening factor of 10 Hz was used in data processing.

### 4.5. Fluorescence Spectroscopy

DAUDA fluorescence measurements were performed using a Jasco FP8200 spectrofluorometer (Jasco, Easton, MD, USA) with a 1 cm path-length quartz cuvette, as described previously [70]. The excitation wavelength was 335 nm (slit width 1 nm) and emission spectra were collected in the range of 350–650 nm. Samples contained 100 µM Tau^4RD^, 300 µM of lipid (OLA or ARA), and 1 µM DAUDA in 10 mM potassium phosphate buffer, 2 mM DTT, protease inhibitors with EDTA, and pH 6.8. All experiments were performed at room temperature.

### 4.6. Far-UV Circular Dichroism (CD) Spectroscopy

CD measurements were carried out with a Jasco J-1500 spectropolarimeter equipped with a Peltier type thermostatized cell holder (Jasco, Easton, MD, USA). Far-UV spectra (190–260 nm) were recorded at 25 °C at a scan rate of 50 nm min^−1^, a bandwidth of 1 nm, and an integration time of 2 s, in 0.1 cm cuvettes. CD spectra were recorded on samples of Tau^4RD^ and αS, in the absence or presence of inducers or lipids. Five spectra accumulations were averaged for each sample, and the spectrum of the buffer was considered as a blank and subtracted. The protein concentration was 6 μM.

To monitor time-dependent spectral changes, samples were incubated in 1.5 mL glass vials at 37 °C under magnetic stirring at 250 rpm. Aggregation reactions were prepared in 700 µL aqueous buffer (10 mM KPi, pH 6.8, 0.5 mM DTT, protease inhibitors with EDTA, 0.02% NaN_3_) and were composed of 20–100 µM Tau^4RD^ or αS, and 300–500 µM of lipid or 25 µM heparin.

Data plots were generated using the software GraphPad Prism 7 (GraphPad Software Inc., La Jolla, CA, USA).

### 4.7. Thioflavin-T Aggregation Assay

Tau^4RD^ solutions were filtered through a 100 kDa cut-off filter (Sartorius Stedim Biotech GmbH, Göttingen, Germany) before starting the aggregation assay to remove pre-existing large oligomers and fibrils. The aggregation was induced by incubating the soluble protein in the presence of FAs at various concentrations (protein:FA was 10 µM:30 µM, 10 µM:300 µM, 100 µM:300 µM) in 10 mM potassium phosphate, pH 6.8, 1 mM DTT. Control reactions were carried out in the presence of heparin (protein:HEP was 10 µM:2.5 µM and 100 µM:25 µM).

The kinetics of aggregation was monitored by measuring the fluorescence of the thioflavin-T (20 µM) added to each sample in a 96-well dark plate (100 µL final volume for each well). Fluorescence measurements were performed using a microplate reader (TECAN Infinite M200 Pro, Tecan Group AG, Männedorf, Switzerland) at 30 °C for ~72 h with cycles of 30 s of shaking (250 rpm, orbital) and 10 min of rest throughout the incubation. The fluorescence intensity was measured every 11 min (excitation, 450 nm; emission, 480 nm; bottom read). Error bars of fluorescence data correspond to standard deviations of at least four independent experiments.

### 4.8. Transmission Electron Microscopy (TEM)

For TEM measurements, samples were prepared as described for the ThT assay at the final volume of 100 µL and incubated for 24 h at 30 °C under intermittent shaking. Subsequently, 30 μL of Tau^4RD^ aggregates (1 μM) in mQ H_2_O were adsorbed onto 400 mesh holey film grid; after staining with 2% uranyl acetate (for 2 min), the sample was observed with a Tecnai G^2^ (FEI) transmission electron microscope operating at 100 kV. Images were captured with a Veleta (Olympus Soft Imaging System, Münster, Germany) digital camera using FEI TIA acquisition software (Version 4.0).

### 4.9. Turbidimetry

Turbidity measurements were performed in a plate reader (TECAN Infinite M200 Pro, Tecan Group AG, Männedorf, Switzerland) by recording absorbance at 350 nm in a 96-well dark plate (100 µL final volume for each well) at 30 °C. Tau^4RD^ or αS (10 µM) were mixed with FA (300 µM) in the polymerization buffer. Error bars in the displayed data correspond to the standard deviations of at least four independent experiments.

## 5. Conclusions

The intrinsically disordered, amyloidogenic protein Tau associates with diverse classes of molecules, including proteins, nucleic acids, and lipids. Evidence is mounting that Tau and lipid molecules are linked in both physiology and pathology, however, both the structural plasticity of Tau and the complexity of lipid states have hindered our thorough understanding of their interactions. Our in vitro study shows that Tau^4RD^, the highly basic four-repeat domain of Tau, associates strongly with ARA and OLA assemblies in a high lipid/protein ratio, perturbing their supramolecular states but itself undergoing structural adaptation in a time-dependent manner. The nature of Tau^4RD^/ARA and Tau^4RD^/OLA co-aggregates appears similar and are therefore not influenced by the degree of unsaturation of the long-chain FA, but aggregates differ from those formed with the detergent SDS. The structural signatures of Tau^4RD^/FA aggregates are further distinct from those of another prototypical IDP, αS, when bound to ARA or OLA, revealing protein-specific conformational adaptations. Both FAs invariably accelerate the self-aggregation of Tau^4RD^ and of αS.

This study highlights new aspects of the Tau^4RD^/FA interactions by providing insights at a sub-molecular level. Although it is not yet clear whether and which lipids form functional co-aggregates in the cellular context, or which are actually involved in neurodegenerative processes, our results contribute to our understanding of the fundamental aspects of Tau/lipid interactions.

## Supplementary Materials

The following are available online. Figure S1: translational diffusion of fatty acids, Figure S2: NMR analysis of ARA micelle formation, Figure S3: turbidity assay, Figure S4: TEM images of FA samples, Figure S5: TEM images of Tau4RD/FA samples, Figure S6: TEM images of Tau4RD/FA samples.
