# Supplementary material for: Unsaturated Fatty Acid-Induced Conformational Transitions and Aggregation of the Repeat Domain of Tau
Source: Molecules. 2020 Jun 11;25(11):2716. doi: 10.3390/molecules25112716 (PMC7321374; doi:10.3390/molecules25112716)
Supplement: Supplementary file 1 [file molecules-25-02716-s001.pdf]

SUPPORTING DATA

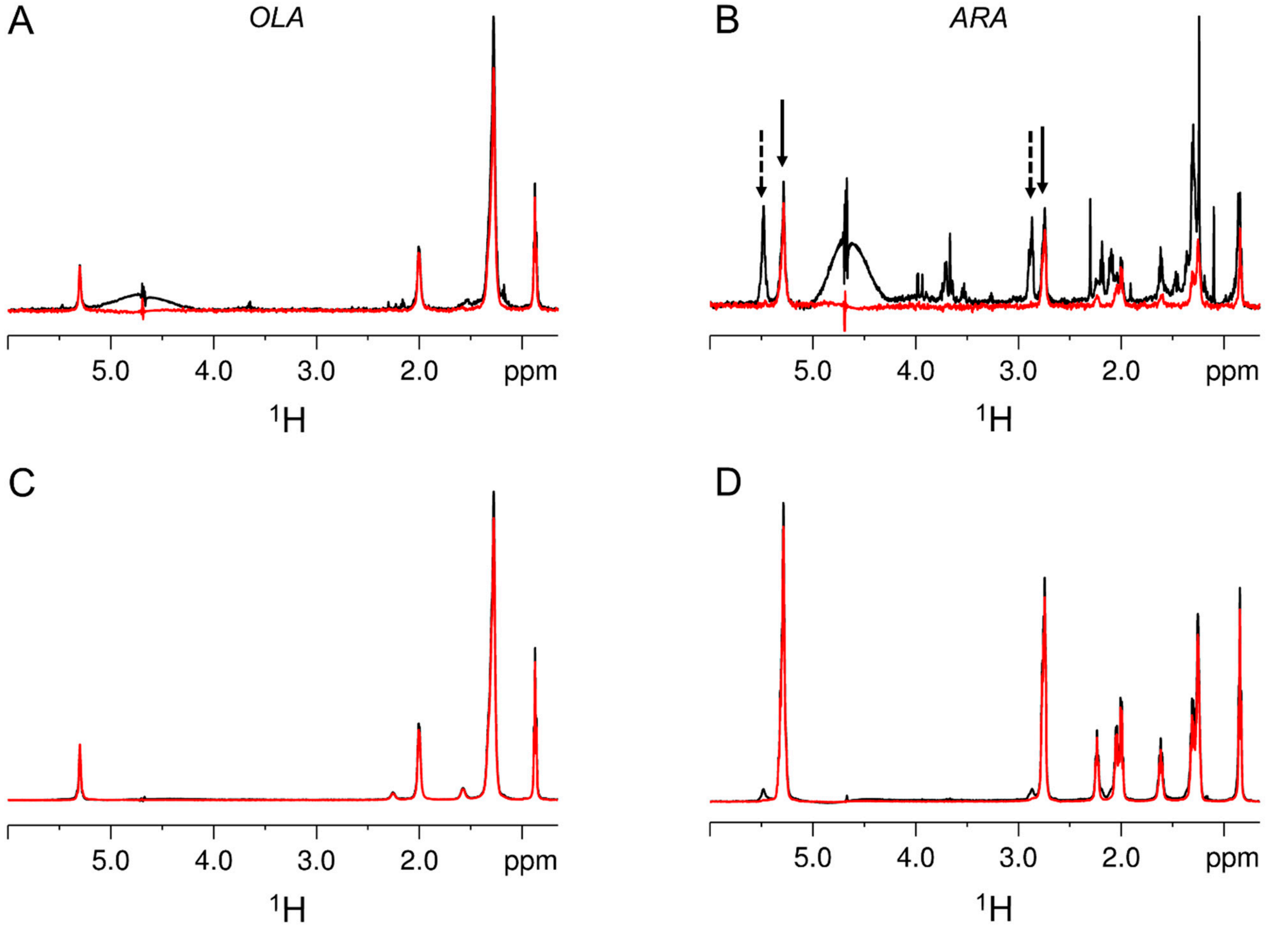

Figure S1. *Translational diffusion of fatty acids*. The displayed NMR diffusion spectra were recorded with 5% (black) and 95% (red) of the maximum gradient strength (53.5 G/cm) and a diffusion delay of 200 ms. The samples contained 75 µM OLA (A), 75 µM ARA (B), 500 µM OLA (C), or 500 µM ARA (D). Peaks indicated by dashed (solid) arrows are assigned to ARA monomers (micelles). The measurement temperature was 37 °C.

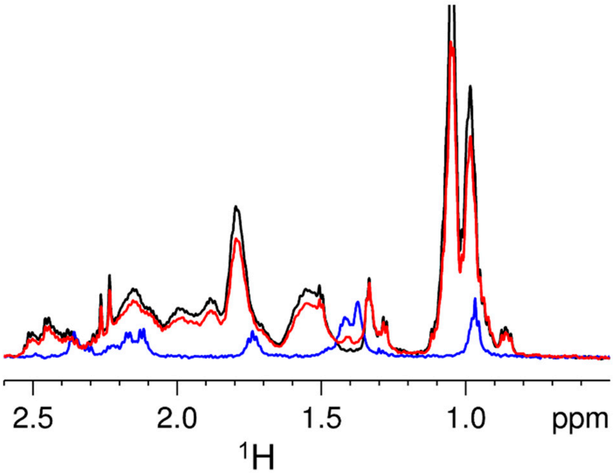

Figure S2. *^{1}H-NMR profiles of Tau^{4RD} and ARA at 20 mM NaCl concentration.* High-field portion of ^{1}H-NMR spectra of 25 µM Tau^{4RD} (black), 75 µM ARA (blue), and 25 µM Tau^{4RD} mixed with 75 µM ARA (red). Measurements were performed at 37 °C.

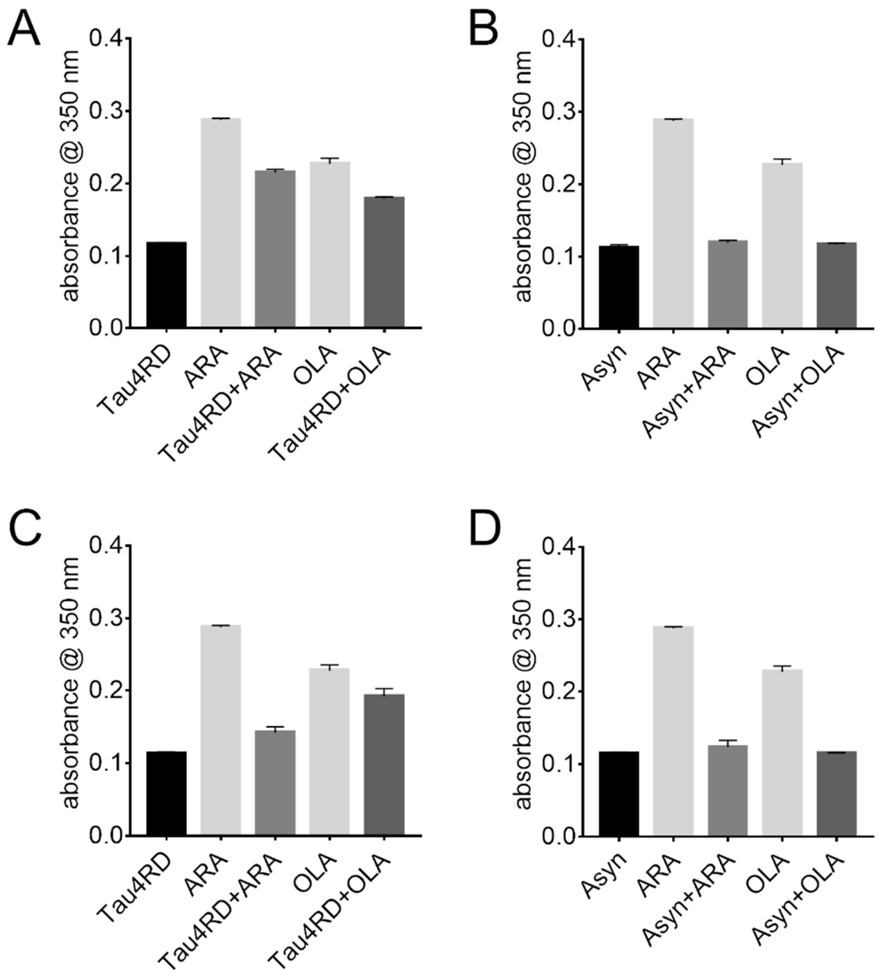

Figure S3. *Turbidity assay.* A,B) Samples contained 10 μM protein and 300 μM FA. C,D) Samples contained 100 μM protein and 300 μM FA. The turbidity was measured at 350 nm and the measurement temperature was 30 °C.

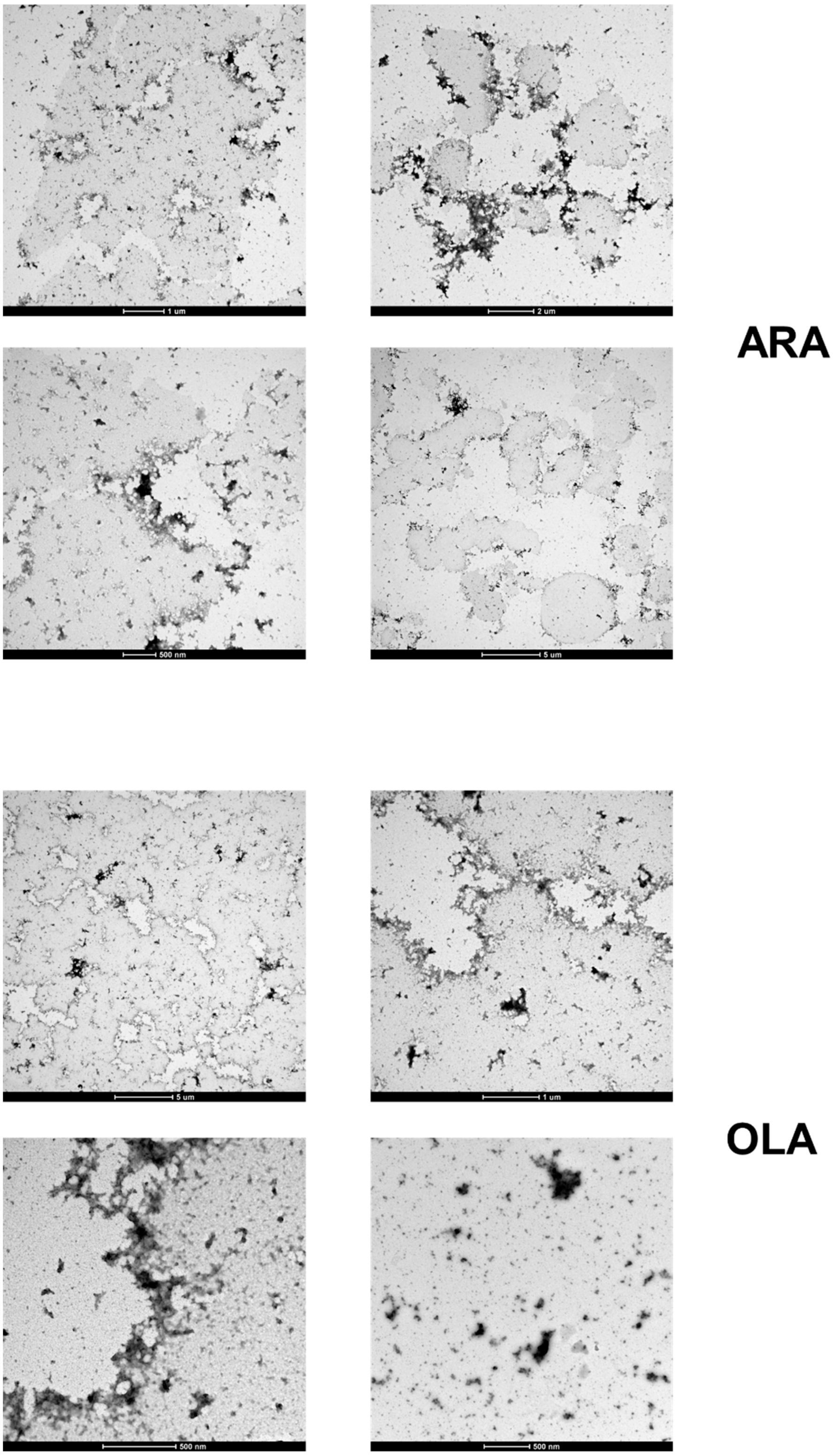

Figure S4. *TEM images of FA samples*. TEM micrographs were taken on FA samples incubated for 24 h at 30 °C under intermittent shaking.

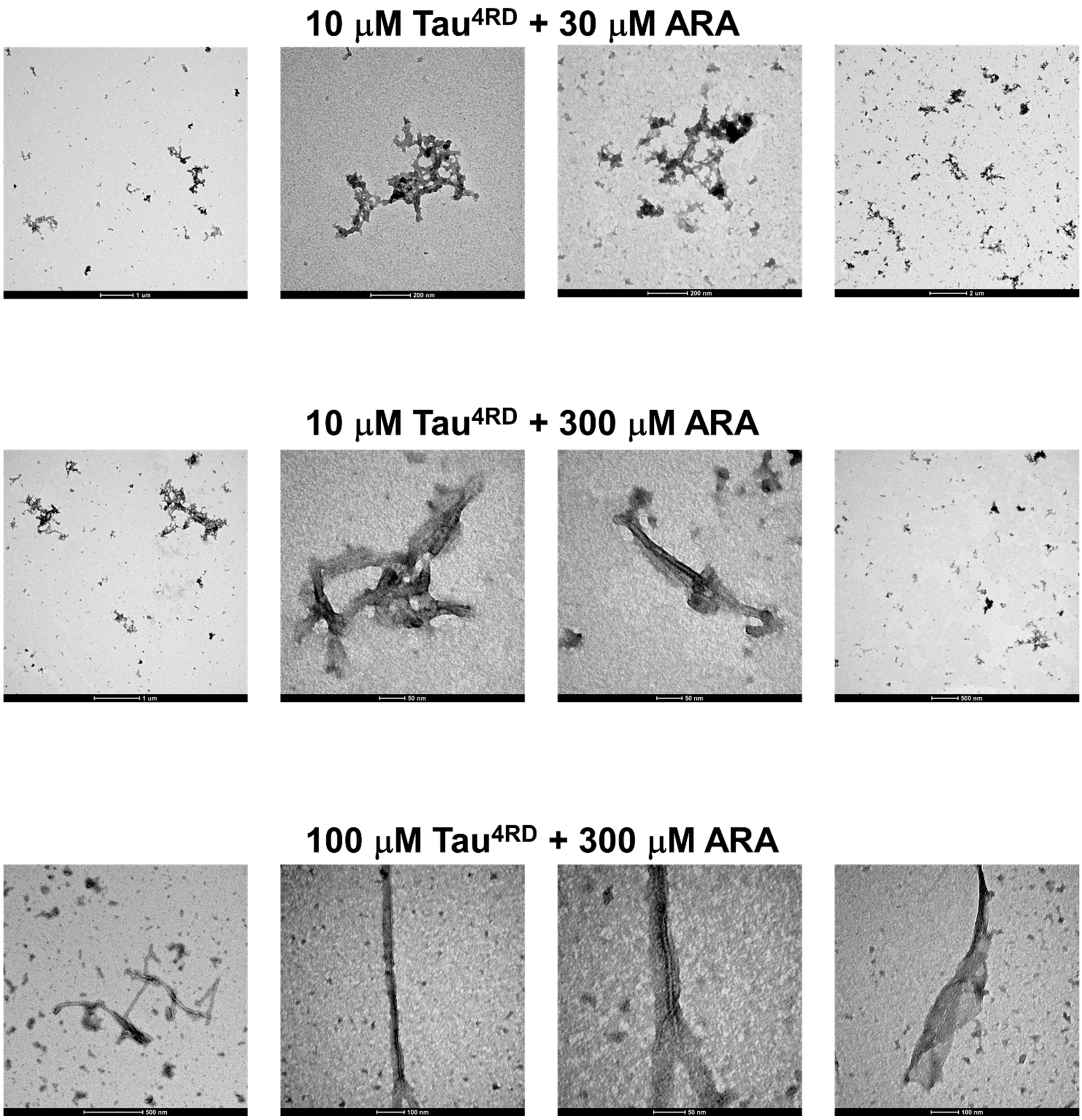

Figure S5. *TEM images of Tau^{4RD}/FA samples*. TEM micrographs were taken on Tau^{4RD}/ARA samples incubated for 24 h at 30 °C under intermittent shaking.

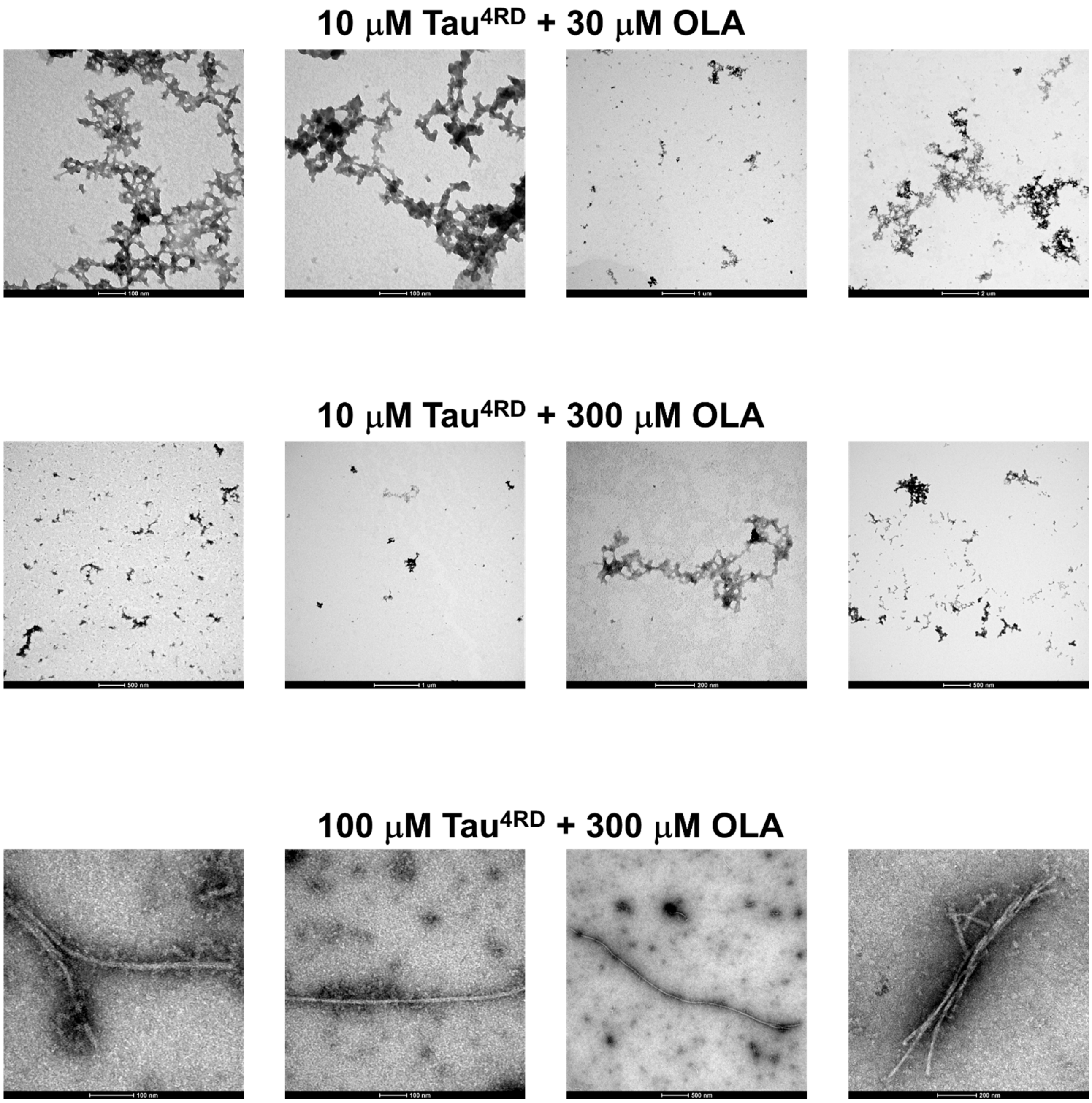

Figure S6. *TEM images of Tau^{4RD}/FA samples*. TEM micrographs were taken on Tau^{4RD}/OLA samples incubated for 24 h at 30 °C under intermittent shaking.
